# KSHV LANA—The Master Regulator of KSHV Latency

**DOI:** 10.3390/v6124961

**Published:** 2014-12-11

**Authors:** Timsy Uppal, Sagarika Banerjee, Zhiguo Sun, Subhash C. Verma, Erle S. Robertson

**Affiliations:** 1 Department of Microbiology and Immunology, University of Nevada, Reno, School of Medicine, 1664 N Virginia Street, MS 320, Reno, NV 89557, USA; E-Mails: tuppal@medicine.nevada.edu (T.U.); scverma@medicine.nevada.edu (S.C.V.); 2 Department of Microbiology and the Tumor Virology Program of the Abramson Cancer Center, Perelman School of Medicine at the University of Pennsylvania, 201E Johnson Pavilion, 3610 Hamilton Walk, Philadelphia, PA 19104, USA; E-Mails: sagarika@mail.med.upenn.edu (S.B.); sunzhig@upenn.edu (Z.S.)

**Keywords:** Kaposi’s sarcoma-associated herpesvirus, KSHV, KS, minichromosome, DNA replication, reactivation, signaling

## Abstract

Kaposi’s sarcoma associated herpesvirus (KSHV), like other human herpes viruses, establishes a biphasic life cycle referred to as dormant or latent, and productive or lytic phases. The latent phase is characterized by the persistence of viral episomes in a highly ordered chromatin structure and with the expression of a limited number of viral genes. Latency Associated Nuclear Antigen (LANA) is among the most abundantly expressed proteins during latency and is required for various nuclear functions including the recruitment of cellular machineries for viral DNA replication and segregation of the replicated genomes to daughter cells. LANA achieves these functions by recruiting cellular proteins including replication factors, chromatin modifying enzymes and cellular mitotic apparatus assembly. LANA directly binds to the terminal repeat region of the viral genome and associates with nucleosomal proteins to tether to the host chromosome. Binding of LANA to TR recruits the replication machinery, thereby initiating DNA replication within the TR. However, other regions of the viral genome can also initiate replication as determined by Single Molecule Analysis of the Replicated DNA (SMARD) approach. Recent, next generation sequence analysis of the viral transcriptome shows the expression of additional genes during latent phase. Here, we discuss the newly annotated latent genes and the role of major latent proteins in KSHV biology.

## 1. Introduction

Kaposi sarcoma (KS) is one of the most common virally induced cancers among HIV-infected patients. KS is an endothelial cell lineage tumor that is caused by Kaposi’s sarcoma-associated herpesvirus (KSHV) or eighth human herpesvirus (HHV-8), sub-classified as a gamma herpesvirus and first discovered in 1994 by Chang and Moore’s group from patients with KS by a subtraction-PCR based method called Representational Difference Analysis (RDA) [1,2]. Clinically, KS exists in several forms; *Classic indolent KS*, which is the prevalent form in HIV-negative elderly male patients of Mediterranean and Middle Eastern origin, *African endemic KS* that is a relatively aggressive form and most common in HIV-negative children, *Iatrogenic KS or post-transplant KS*, associated with patients undergoing immunosuppressive therapy after renal transplantation, and *AIDS-related epidemic KS*, which develops in HIV-infected individuals and is among the leading causes of death in AIDS patients [3]. Though KSHV is found in all forms of KS, infection with KSHV is necessary but not sufficient for the development of the KS [4,5,6].

After the discovery of KSHV in KS tissues, KSHV sequences were rapidly identified in two other lymphoproliferative disorders: (a) Primary Effusion Lymphoma (PEL) and the (b) plasmablastic variant of Multicentric Castleman’s Disease (MCD) [7,8]. Spindle cells expressing endothelial markers including CD31, CD34, CD36 and factor XIII are shown to be the cell type of origin in KS, whereas PEL and MCD are considered to be of B-cell lineage as they express B-cell surface markers. Additionally, KS and MCD are polyclonal in nature while there is a pattern of B-cell monoclonality in all PEL samples [9,10]. In immunocompetent individuals, CD19+ B cells appear to be the main target of KSHV infection [11]. Recently, a newly characterized KSHV-associated condition, KICS (KSHV Inflammatory Cytokine Syndrome) has been reported in patients with HIV and KSHV co-infection with elevated levels of interleukin-6 and KSHV, though the cell origin of this condition is still a matter of discussion [12]. Although the incidence of KS in HIV-infected individuals has declined with the introduction and widespread use of highly active antiretroviral therapy (HAART), it is anticipated that KSHV will remain one of the co-morbid agent in persons with or at high risk of acquiring HIV infection [13]. Therefore, understanding the molecular pathogenesis of KSHV infection and KSHV-HIV co-infection is crucial for development of drug therapies to treat and control the associated pathological processes.

## 2. Epidemiology

Serologic and epidemiologic studies have revealed that KSHV does not lead to a ubiquitous infection and KSHV-infected individuals are found throughout the world. There is an interesting but enigmatic association between KS prevalence and KSHV seroprevalence, though there are major variations in KS prevalence geographically [14,15].

The advent of serological assays to detect antibodies against KSHV has enabled the study of the distribution of KSHV seroprevalence among different risk groups. Currently, in the most frequently used serological assays such as immunofluorescence assay or ELISA, serum antibody to KSHV is detected using either KSHV-infected cells or recombinant capsid proteins/KSHV lysate as antigens, respectively [16,17,18]. Among the general population, the seroprevalence of KSHV infection in northern Europe, Asia and America is <10%, but the overall KSHV seropositivity is >50% in most of sub-Saharan Africa. The Mediterranean regions have intermediate seroprevalence that ranges from 30% in Sicily to 3% in northern Italy [19].

Genotypes of KSHV are categorized based on highly variable but tracer K1 and K15 genes located next to the terminal repeat in the viral genome. As a result, analysis of the divergence of the K1 gene has led to the identification of 5 genotypes (A through E) and several subtypes [20,21]. In general, the sequence variation between different subgroups is <3% at the nucleotide level, however two hyper-variable K1 regions-VR1 and VR2 display up to 60% variability [22]. Subgroups A1-4 and subtype C are prevalent in North Europe, America, some regions of Asia and Middle East and subtype A is predominant in AIDS-associated cases but not in non-AIDS patients. Subgroups B1-4 are primarily found in sub-Saharan Africa whereas subgroups D and E are abundant in Australia, the Pacific and Brazilian Amerindians [23,24].

Although the transmission modes and the risk factors of KSHV have not been clarified yet, both sexual and non-sexual modes can transmit KSHV [25,26]. In KSHV-endemic regions like Africa, saliva-mediated transmission is the most common mode of transmission among children, as high KSHV copy numbers are detected in the saliva of seropositive patients while sexual transmission may be predominant among homosexual men in the areas of low prevalence where the risk of KSHV transmission rises with the number of sexual partners [27,28]. Organ transplantation can also transmit KSHV and cause viral infection and is documented to occur both in infected organ donor and transplant recipients due to virus reactivation [29,30]. KSHV transmission through blood transfusion or among intravenous drug users is rare but evidence of both modes of transmission has been reported [31].

## 3. Life Cycles of KSHV and Control of Latency

Full genome sequence of KSHV virus from KS lesions and PEL cells reveal that the viral genome exists as a linear double-stranded DNA of *ca.* 170 kb in the virion particles. The genome consists of a central ~137 kb long unique coding region (LUR) with 53.5% GC content, which is flanked by multiple, non-coding terminal repeat units having 84.5% GC content [32]. The number of repeats in the TRs varies from 32–50 copies [33]. LUR is the viral protein-coding region, which encodes approximately 90 ORFs, (some with homology to the cellular genes), 12 microRNAs and several ncRNAs [34]. Usually the complete viral particle consisting of a viral capsid and an envelope, 150–200 nm in diameter, is not observed in the KS samples by electron microscope, as the genome is maintained in latent form without reactivation [35]. However, they can be observed in PEL cells induced by Na-butyrate (NaB) or 12 *O*-tetradecanoylphorbol-13-acetate (TPA) [36]. Following infection, viral DNA is delivered to the nucleus where it is circularized using host enzymatic machinery, generating a chromatinized nuclear episome [37]. Studies to identify the processes through which the incoming histone-free virion DNA gets chromatinized in the nucleus are still in its infancy.

Like the other members of the γ-herpesvirus family, KSHV exhibits two different phases of infection: *persistent latent infection* and a *transient lytic reactivation* that are distinguished by their viral gene expression patterns. During latent infection, which is the default pathway of KSHV infection *in vivo* and *in vitro*, the viral genome is maintained as a circular episome within the host cell nucleus with highly restricted protein expression in order to maintain the genome in the dividing cells and to limit host immune responses while enhancing cell survival and virus persistence [38]. The latent phase is reversible and certain environmental and physiological factors including oxidative stress, hypoxia or inflammatory cytokines may periodically reactivate the hidden latent virus to enter the second program of viral gene expression and lytic reactivation [39,40,41,42,43]. In this phase, expressions of the remaining viral open reading frames (ORFs) are activated in a temporally regulated cascade, leading to three classes of lytic genes: immediate early (IE), early (E) and late (L) genes [44]. The host cell machinery is redirected to manufacture and assemble the infectious progeny virions, ultimately causing cell apoptosis due to virus production and shut off of host cell protein synthesis. In KS tissue, KSHV is found predominantly in the latent phase as assessed by immunohistochemical and genetic analysis of KS tissue, while there is limited expression of KSHV viral genes especially vIL-6 in PEL cells. In contrast, MCD is associated with KSHV in its lytic state, a feature that is unusual among herpesvirus-associated tumors [45].

The dogma of the classic transcriptional program shifted to a more complex expression pattern after technological advances enabled the discovery that KSHV induces diverse gene expression profiles in different endothelial cell types, lymphatic endothelial cells (LECs) and blood endothelial cells (BECs) [46]. The gene expression pattern analyzed in these cells infected with recombinant virus rKSHV.219 clearly demonstrated that infected BECs exhibit conventional latent gene transcripts during latency, whereas LECs have a unique virus latency program with a widespread expression of numerous lytic genes, including RTA, K-bZIP and ORF45 that do not produce any infectious virions [46,47]. During this “dysregulated lytic program”, expression of KSHV ORF45 leads to selective activation of mTORC1 by ERK2-mediated activation of RSK1, which sensitizes these LECs to rapamycin-induced killing. However, the exact benefits of this different transcriptional program to KSHV during persistent infection are yet to be identified.

Recent analysis of the KSHV genome by Arias *et al.* using next-generation sequencing, mRNA-Seq and Ribo-Seq has provided a plethora of information about the genomic landscape and peptide coding potential of KSHV during the productive (lytic) cycle [48]. Using a tightly-controlled and highly inducible epithelial iSLK-219 cell line, several hidden genomic and functional features have been uncovered, leading to the generation of a novel revised annotation of the KSHV genome: KSHV 2.0 [48]. This includes 45% more coding capacity with expanded illustration of 49 viral transcripts, 70 ORFs, non-coding RNAs, polyadenylation sites, splice junctions and initiation/termination codons of main ORFs [48]. The coding capacity of KSHV has been attributed to multiple strategies, including splicing, mRNA editing and the usage of alternative start sites leading to multiple small and upstream ORFs (sORFs and uORFs) [48]. In summary, these comprehensive and high resolution approaches have identified the underappreciated complexity of the KSHV gene expression during the different stages of the viral life cycle [48].

Central to KSHV infection is the ability of the virus to establish life-long, non-productive latent infection, which can later be reactivated in the host cell. In general, this oncogenic γ-herpesvirus faces a number of problems to establish and control the latency in the infected host cell without losing the genome [49]. To begin with, the incoming epigenetic naïve and linear KSHV genome must be circularized and chromatinized following infection, to generate the KSHV epigenome and trigger the transcriptionally silent latent cycle. Further, one or more of the expressed latent genes must allow stable latent dsDNA replication and KSHV genome segregation to maintain the genome in new daughter cells. Additionally, successful latency establishment also requires hijacking of several viral and cellular pathways in order to repress KSHV reactivation and to escape host immune surveillance. Thus, KSHV’s existence and persistence in the host cell requires a dynamic balance between the two gene expression programs, although how this balance is maintained still remains unclear. Here we discuss in detail the latent maintenance of the dsDNA KSHV genome in the infected host cell with particular attention to the role of one of the major latent antigens, latency-associated nuclear antigen (LANA) which is constitutively expressed in all latently infected KSHV cells and is an important contributor to efficient KSHV transcriptional regulation.

## 4. The Latency Program of KSHV and the Key Players

**Figure 1:**
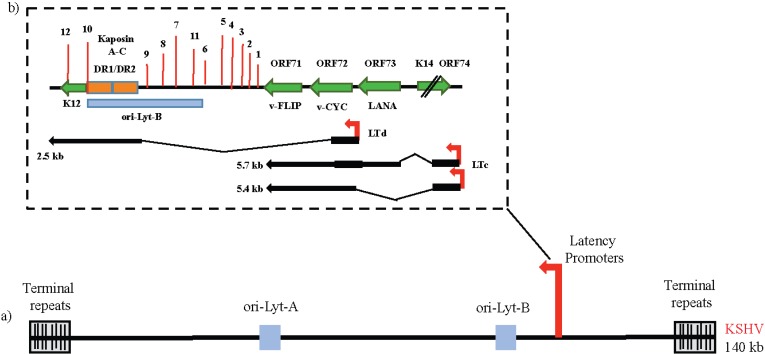
(**a**) Schematic representation of the linear KSHV genome with terminal repeat sequences, origin of lytic replication (ori-Lyt-A and ori-Lyt-B) and latency promoter; (**b**) The major latency locus of KSHV is shown in an expanded view. **Top** panel: The four major open reading frames (ORFs)-ORF73/LANA, ORF72/v-CYC, ORF 71/v-FLIP and ORF K12/Kaposins A-C along with the adjacent K14 and ORF74 genes are shown by green arrows. Position of 12 pre-miRNA sequences is shown as vertical red lines. **Bottom** panel: The schematic diagram of the transcripts directed by the Kaposin promoter (LTd) and the LANA promoter (LTc) is shown.

Examination of latent infection in PEL cell lines that maintain 98%–99% of latently infected cells has led to the characterization of a major latency locus that is abundantly and consistently transcribed in cell lines derived from the KSHV-infected patients and restrict transcription to only a few of the 90 KSHV genes (Figure 1) [50,51]. This latency locus includes four genes, encoding ORF73/LANA, ORF72/v-Cyclin, ORF71/v-FLIP (*Fas-associated death domain-like interleukin-1β-converting enzyme-inhibitory protein*) and K12/Kaposin family of protein (Kaposin A, B and C) along with 12 microRNAs that can be processed to yield 18 miRNAs (at last count) [52,53,54]. Viral interferon regulatory factor-3 (v-IRF-3) and mRNA of several other viral genes including viral G protein-coupled receptor, vGPCR encoded by ORF74, K14, vIL-6 and Processivity factor encoded by ORF59 have also been detected in most KS and PEL-tumor models [55]. Together, these latency-associated viral proteins are required for constant latent infection and survival of the infected cell. Latency transcript cluster, including, LANA, v-Cyclin and v-FLIP are located adjacent to each other and are transcribed from a constitutively active promoter, LANA promoter or LTc [49]. These three genes are separated from the K12 gene which is transcribed from a second promoter, the kaposin promoter, or LTd, located just downstream of LANA. The kaposin promoter encodes a spliced transcript encoding the kaposin proteins and a bicistronic RNA for v-Cyclin and v-FLIP [56]. This promoter also governs the expression of 12 KSHV pre-miRNAs. The ORF74-K14 transcript initiates from 5' UTR of the LANA-vCyclin-vFLIP and is expressed in latently infected cells along with LANA-vCyclin-vFLIP genes expression.

Another unlinked latency promoter is found to encode the vIRF3 gene, a member of IRF superfamily [49]. This protein has been identified as the LANA-2 protein encoded by the ORF K10.5 of KSHV and is expressed in KSHV-infected hematopoietic tissues including PEL and MCD, but not in KS spindle cells. This protein is known to inhibit the function of certain cellular IRFs and block the interferon induction. Wies *et al.* have shown that vIRF3 is also required for proliferation and survival of PEL cells infected with KSHV as *in vitro* knockdown of vIRF3 expression in PEL cells reduced the cell proliferation and increased the activity of caspase-3 and/or caspase-7, triggering programmed cell death [57].

Additionally, a third latency locus has been identified which drives the expression of the ORF-K1 protein [58]. K1 is a 46-kDa transmembrane signaling protein that imitates signaling through the B cell receptor [59]. In many KSHV-infected cell lines, this gene is transcribed at very low levels during latency and is upregulated during lytic reactivation [60]. More sensitive methods using microarrays and proteomics have identified several viral transcripts and peptide motifs that further provide valuable knowledge regarding viral latent gene expression. In the following sections, we describe some of these key KSHV latent genes and their role in KSHV-associated latency.

### 4.1. ORF73/LANA (Enables Replicative Immortality)

As mentioned above, KSHV establishes stable latent infections that play an essential role in KSHV-induced malignancies and pathogenesis. One of the key aspects of KSHV-associated oncogenesis is the ability of the latent viral genome to persist as extra-chromosomal episomes in the dividing cells that express a dynamic pattern of viral genes to drive host cell proliferation and survival. As the KSHV viral genome does not encode its own centromeric proteins, it is probable that it follows an alternative way to maintain and replicate its episome from parental cells to progeny cells. In KSHV, LANA, a major KSHV-encoded latent protein is considered to be critical for the maintenance, replication and efficient segregation of the viral genome from generation to generation. In order to carry out this function, LANA, a multifunctional nuclear protein (1162 amino acid in length and 220–230 kDa in size), binds directly to the conserved TR sequences of the KSHV genome through its *C*-terminal domain and docks onto the host chromosome through its *N*-terminal chromatin-binding domain (CBD), thus enabling the KSHV genome to hitch a ride on the host chromosome during mitosis and maintain a stable copy number in the latently infected cells. Also, among all the latent proteins, LANA is the most consistently expressed antigen in the KSHV-infected cells and is always detected as a dot-like staining pattern by immunohistochemistry. LANA is found to bind and interact with multiple cellular proteins, including *tumor suppressors*-p53, pRb and von Hippel Lindau (VHL), *transcription factors*- ATF4/CREB2 and STAT3, *chromatin-binding proteins*-HP1, H2A/H2B, MeCP2, Brd4 as well as *signal transducers*-GSK-3β, in order to inhibit apoptosis and stimulate spindle cell proliferation. LANA has also been proposed to bind to several viral promoters and suppress viral lytic gene transcription and thereby influence the maintenance of latency. Thus LANA is a highly versatile oncogenic protein that plays a central role in the pathogenesis of KSHV infection.

### 4.2. ORF 72/*v-*Cyclin (Sustains Cell Proliferation)

ORF72 encodes the functional viral homologue of cellular cyclin D. Like its cellular homologue, v-Cyclin acts like a constitutive activator of cellular cyclin-dependent kinase 6 (CDK6) and regulates cell cycle and cell proliferation [61]. The v-Cyclin mediates phosphorylation and inhibition of its cellular counterpart pRb protein, Histones H1, CDK inhibitor (cdki) and p27 (Kip1) through the formation of v-Cyclin-CDK6 complex [62]. The exact role of this viral protein in regulating KSHV life cycle is not fully understood but studies indicate that v-Cyclin mediates phosphorylation of nucleophosmin (NPM), through its association with CDK6 and facilitates NPM-LANA interaction and recruitment of HDAC1 to promote KSHV latency [63]. Additionally, due to its close functional relationship with murine gammaherpesvirus 68 (MHV68) v-Cyclin, KSHV v-Cyclin is believed to function as a modulator of the latent-lytic switch [64].

### 4.3. ORF71/*v-*FLIP (Resists Cell Death)

*ORF71* encodes the KSHV homologue of cellular FLICE (Fas-associated death domain (FADD)-like interleukin-1 beta-converting enzyme) inhibitory protein, v-FLIP or K13. The v-FLIP activates one of the key cellular survival pathways, the NF-κB pathway, in latently infected PEL cells to promote cell proliferation and survival during latency [65,66]. It does so by binding to the inhibitor of kB-kinase γ (IKK γ) thereby leading to the activation of the NF-κB pathway [67,68]. NF-κB pathway activation by v-FLIP has been linked to KSHV lytic replication as a KSHV mutant lacking the v-FLIP gene is shown to inhibit ORF 50/RTA lytic gene expression [69].

### 4.4. K12/Kaposins

The Kaposin locus located a few kilobases away from the v-FLIP gene, upstream of the ORF73 is a complex and poorly understood locus that encodes for at least three proteins, namely, Kaposin A, B and C [54]. Kaposin A is a small hydrophobic latent oncogenic protein with inefficient transforming potential in rodent fibroblasts whereas Kaposin B is a small soluble nuclear protein, which acts like a scaffold or adaptor protein and activates p38/MAPK signaling pathway after binding to a key p38 substrate called mitogen-activated protein kinase 2 (MK2) [70]. All these proteins are shown to contribute to the pro-inflammatory KS tumor microenvironment.

### 4.5. Viral microRNAs

As shown in Figure 1, the kaposin locus also encodes 12 pre-miRNAs, out of which 10 miRNA (miR-K1-9 and -K11) are encoded between kaposin and the ORF71/K13 region while the other two miRNAs, miR-K10 and -K12 are located in the coding and 3' untranslated region of K12, respectively [71]. These KSHV-encoded pre-miRNAs further produce 18 mature miRNAs that are highly conserved in all the KSHV isolates. All miRNAs are expressed in latently infected cells and deletion of most viral miRNAs leads to a modest enhancement of KSHV reactivation, suggesting an important role of these miRNAs in regulating latent gene expression [72,73]. Among these miRNA, miR-K1 represses the expression of IκBα-an inhibitor of the pro-survival NF-κB pathway and inhibits the activation of lytic viral promoters. KSHV miR-K10 affects the TNF-like weak inducer of apoptosis (TWEAKR) and inhibits cell apoptosis by suppressing pro-inflammatory responses, which might contribute to KSHV latent infection [74]. Several other miRNAs such as miR-K3, -K4, -K5 and -K9 target nuclear factor I/B, Rbl2 protein, Bcl-2 associated factor, BCLAF1 and activates 3'UTR region of RTA protein respectively, in order to induce viral reactivation. As these miRNAs are expressed in latency, they could potentially target both cellular and viral miRNAs and contribute to the neovascular phenotype of KS [75].

## 5. Role of LANA in KSHV Episome Maintenance and Partitioning during Cell Division

In latently infected cells, the KSHV genome persists as an episome, a closed circular extra-chromosomal genome. In each KSHV-infected PEL cell, the copy number of the KSHV episome seems to be stable (~50–100 copies/cell) [76,77]. To accomplish this, the KSHV episome must replicate and then efficiently segregate to the daughter cells after mitosis to avoid the loss in copy number. During mitosis, the replicated viral episomes are segregated to the daughter cells mediated by LANA. LANA is a multifunctional protein required for episomal maintenance and segregation as deletion of KSHV LANA resulted in the complete loss of episomal genomes and failure to establish latent infection [78,79]. To successfully partition the episomes into dividing daughter cells, LANA simultaneously binds to the viral episome at the TR region and the host chromatin [80,81]. The *C*-terminal domain of LANA binds cooperatively to two sites (LANA binding sites) within each TR element, which is necessary for episome persistence and efficient segregation of KSHV episomes to the daughter cell [82,83]. In KSHV infected cells, LANA tethers to the condensed mitotic chromatin through interactions with several host proteins. These include H2A/B, histone H1, MeCP2, Brd4, NuMA, Bub1 and CENP-F [84,85,86,87,88,89,90].

A condensed chromatin consists of a DNA double helix wrapped around core histones (nucleosomes) compacted by linker histone (H1) along with other nucleosomal proteins. The histone proteins are the chief protein components of chromatin, acting as spools around which DNA winds. Histones are a family of small, positively charged proteins termed H2A, H2B, H3, and H4 and H1. The histones H2A, H2B, H3 and H4 are the core histones, while histone H1 is the linker histone. Two molecules each of H2A, H2B, H3, and H4 form the histone octameric nucleosome, which is bound and wrapped with DNA [91]. The linker histone H1 binds the nucleosome at the entry and exit sites of the DNA, thus locking the DNA into place and allowing the formation of the higher order structure [92]. It has been reported that LANA interacts directly with chromosomes via histones H2A and H2B (H2A/B) in the cells and uses them as a docking station to tether viral episomes to the cellular chromatin. By using affinity purification and mass spectrometry, Barbera *et al.* found that the *N*-terminus of LANA (LANA 1-32) can bind to the core histone proteins, H2A, H2B, H3 and H4 as well as Ku70, Ku80 and PARP1. The metaphase spread assays performed in knockout mouse embryonic fibroblasts (MEFs) showed that Ku70, Ku80, PRAP1 do not mediate the association between LANA and the chromosome. Since nucleosomes are comprised of two H2A/H2B dimers and one H3-H4 tetramer wrapped by DNA, LANA binding to any of the histone components would result in precipitation of all four core histones of the nucleosome, therefore further studies using immunoprecipitation and GST pull down assays showed that the *N* terminus LANA 1-32 and the full-length LANA can both specifically bind to H2A and H2B rather than H3, H4, and this binding can be found throughout the cell cycle. The roles of H2A/H2B in LANA-host chromosome association were further confirmed in *Xenopus laevis* sperm chromatin, which is naturally devoid of H2A/H2B. These experiments showed that LANA cannot bind to *Xenopus laevis* sperm chromatin, but this chromatin binding can be rescued after assembly of nucleosomes containing H2A/H2B [84].

The structure of LANA bound to the nucleosome resolved by X-ray diffraction showed that the *N*-terminus of LANA directly binds nucleosome core particles; the nucleosomal surface functions as a docking station for LANA [84]. Also a hairpin formed by the KSHV *N*-terminus of LANA is seen to interact with host chromatin through binding to an acidic patch formed by H2A/H2B dimer within the nucleosome. Analysis of the molecular surfaces of both, the LANA peptide and the H2A-H2B dimer demonstrate an appropriate charge complementarity, indicating that the LANA *N*-terminal region has evolved to recognize this region of the nucleosome core particles with high specificity [84]. It is very interesting that LANA cannot bind to either H2A or H2B alone, but is found to be associated with the H2A/H2B dimer [93]. Besides the H2A/B dimer of the nucleosome core particles, the association between LANA and the linker histone H1 was also found in body cavity-based lymphoma (BCBL) cells [90]. A recent report showed that LANA can associate with H2AX, an isoform of H2A, to contribute to the persistence of the KSHV genome in KSHV-positive cells [94]. These results strongly suggested that H2A/H2B is not only important but also essential for LANA chromosome association.

The centromere is a chromosomal apparatus that is required for chromosome segregation by ensuring the delivery of one copy of each chromosome to each daughter at cell division [95]. In mitosis, some kinetochore proteins such as Bub1 and CENP-F assemble at the surface of centromere and act as the docking site of the spindle microtubule binding [96]. As viral genomes lack centromeres, the KSHV episomes must tether to the host chromosomes to ensure that they are partitioned to daughter cells. A recent report showed that KSHV achieves this through LANA, which simultaneously binds to KSHV viral genomes and the centromeres of mitotic chromosomes via the formation of complexes with CENP-F and Bub1 [89]. Both the *N*-terminal and *C*-terminal domains of LANA strongly bind to Bub1 and CENP-F. The *N*-terminus of LANA showed greater efficiency of binding to CENP-F, whereas the basic *C*-terminal domain of LANA has a minor binding affinity to CENP-F as compared to the *N*-terminal domain. The dynamic association of LANA and Bub1/CENP-F demonstrated by immunofluorescence assays showed strong co-localization of LANA and Bub1 at each phase during mitosis and interphase. Also, the co-localization of LANA, Bub1 and the KSHV episomes was shown using fluorescence *in-situ* hybridization (FISH) assay. A dramatic decrease in the copy number of KSHV episomes was found in Bub1 knockdown KSHV-infected cell lines [89]. However, no significant changes in the KSHV episome copy number was observed in CENP-F1 knock-down KSHV positive cell lines [89]. The Bub1 knockdown KSHV-positive cells showed significant reduction in the number of KSHV episomes, which may be due to an inefficient passage of KSHV episomes to the progeny nuclei during mitosis and failure to maintain the viral genome in the absence of Bub1. This suggests that the interaction of CENP-F with LANA is probably redundant, compared to Bub1, although important in the context of LANA’s function to tether KSHV genome to the host chromosome to ensure persistence of the viral genome. As a component of the spindle assembly checkpoint (ASC), Bub1 is important for the formation of mitotic checkpoint complex (MCC) [97,98]. The interaction of LANA with Bub1 might interfere with the function of Bub1 and the correct spindle formation, although this interaction is critical for segregation into the daughter cells. A recent study showed that LANA can promote the degradation of Bub1 in a ubiquitin dependent pathway which finally resulted in chromosomal instability (CIN) in KSHV-infected tumor cells [99].

Nuclear mitotic apparatus (NuMA) protein (238-kDa in human) is a component of the mitotic apparatus used for the segregation of dividing nuclei [100,101]. During the metaphase and anaphase stage of mitotic cell division, NuMA is distributed to the spindle poles to organize microtubule movement and to stabilize the mitotic spindle [102,103,104]. NuMA has also been shown to associate with small nuclear ribonucleoproteins and splicing factors involved in recycling and phosphorylation of RNA-processing factors and thus has been implicated in the regulation of DNA replication and transcription [105,106,107]. As a nuclear matrix protein, NuMA is thought to support the nuclear shape in differentiating cells [108]. In addition, a role for NuMA in DNA anchoring has also been proposed based on its interaction with matrix attachment regions, which anchor DNA on the nuclear matrix [109]. NuMA is also known to interact with a number of essential mitotic components, including microtubules [110,111], dynein/dynactin [112] and has crucial functions related to cell cycle progression [101,109,113,114]. NuMa has been shown to play a vital role in the maintenance of KSHV genome in the host cell in a cell cycle dependent manner [85]. During the interphase, NuMa may serve as the nuclear matrix and support the KSHV genome maintenance and segregation into the new daughter cells. We have shown that NuMA and LANA can interact with each other during interphase and their interaction is temporally lost as the cells enter the mitotic phase [85]. During mitosis, NuMA forms complexes with dynein/dynactin and microtubules, which is important for the segregation of replicated viral DNA. It has been shown that the amino and carboxy-terminal domains of LANA have different functions and can co-operate to tether KSHV genome to the human chromosomes [115]. It has been reported that NuMA binds to LANA in the carboxy-terminus between amino acids 840 and 963, which is adjacent to the TR-binding region and also involves interactions with dynein/dynactin and microtubules [85]. Additionally, the involvement of dynein/dynactin and microtubules in this interaction was proved by blocking the association of NuMA with these proteins, which resulted in the loss of KSHV episomes in the daughter cells.

Methyl CpG binding protein 2 (MeCP2) is a nuclear protein of about 75–80 kDa, that preferentially binds to methylated CpG dinucleotides [116,117,118]. It has been suggested that MeCP2 plays a key role in the transcriptional silencing of genes in CpG-methylated regions, activation of euchromatic genes, and mRNA splicing [119,120,121]. MeCP2 may also alter higher-order chromatin architecture [122,123]. It has been shown that MeCP2 interacts with the *N*-terminus of LANA and this interaction is vital for tethering of LANA to the host chromosomes [124]. In a separate study, it has been reported that the association of LANA and MeCP2 is modulated by the chromatin-binding motif of LANA located at the *N*-terminus [86]. In the same study, the authors also reported that co-expression of fluorescently tagged LANA and MeCP2 in murine cells resulted in remarkable relocalization of LANA from its diffuse nucleoplasmic distribution to being concentrated at the chromocenters, which corresponds to major accumulations of pericentric heterochromatin. Although the methyl-CpG-binding domain in LANA is sufficient to localize MeCP2 to chromocenters, relocalization of LANA requires both the methyl-CpG-binding domain and transcription repression domain. MeCP2 has been reported to enhance the transactivation of human E2F1 promoter through LANA and this effect is dependent on the chromatin-binding motif and methyl-CpG-binding domain. These findings indicate that multiple interactions are required for LANA to stably associate with chromatin and may occur as a two-step process in which nucleosome binding by the chromatin-binding motif facilitates the interaction of LANA with sequence- or context-specific cofactors such as MeCP2. These multivalent interactions possibly allow LANA to stabilize MeCP2 on low-affinity sites and facilitate KSHV to reprogram selected aspects of host gene expression [86].

DEK is a ubiquitous nuclear protein of ~43 kDa and has been shown to predominately associate with chromatin [125]. The protein was first identified in a chromosomal translocation with the NUP214 nucleoporin protein in a subset of acute myeloid leukemias [126]. It is also a ubiquitously expressed DNA-binding phosphoprotein that recognized Ets-binding sites in the human immunodeficiency virus type-2 enhancer [127,128,129], and is a constituent of splicing complexes [130]. It has been shown that DEK interacts with the carboxy-terminal domain of LANA between the amino acids 986 to 1043. Unlike the punctate localization of LANA on chromosomes of infected cells, DEK has broad distributions on human chromosomes [124]. It has been suggested that DEK associates with histones and plays a supporting role in LANA-mediated tethering to the host chromosome [131].

**Figure 2:**
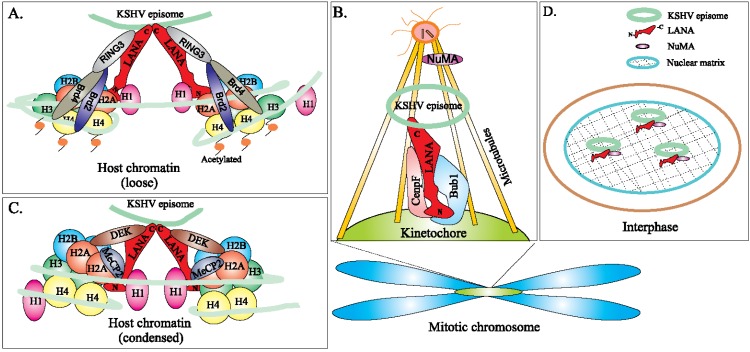
Schematic model showing the association of LANA with host proteins that aid in tethering to the host chromatin and segregation. The interaction of host cellular proteins with LANA during interphase (panel **D**) and the Mitotic phase (M-phase) is shown. During interphase, NuMA binds to the nuclear matrix and tethers KSHV genome by interacting with the carboxy-terminus of LANA, whereas during the M-phase, this interaction is lost and NuMA interacts with the microtubules and localizes to the spindle poles. Panel **A** shows the condensed chromatin structure during M-phase. LANA interacts with the core histone proteins (H2A, H2B, H3 and H4) through the *N*-terminal domain and binds to the KSHV episome through the *C*-terminal domain. Nucleosome binding by the chromatin-binding motif facilitates interaction of LANA with MeCP2 at the *N*-terminal domain of LANA. DEK protein interacts with the *C*-terminal domain of LANA and also to histones H2A, H2B, H3 and H4 facilitating tethering of LANA to the host chromatin. Panel **B** shows that both the *N*- and *C*-terminus of LANA strongly bind to the kinetochore proteins Bub1 and CENP-F that ensures delivery of KSHV episomes to the daughter cells during chromatid segregation. Panel **C** shows a loose chromatin structure, where BRD2/RING3 interacts with acetylated histone H4 and BRD4 interacts with acetylated histones H3 and H4, whereas LANA binds to the RING3 through the *C*-terminal domain, thus showing the contribution of host BET proteins in KSHV genome persistence.

Bromodomain and extra-terminal (BET) proteins are a class of highly conserved bromodomain (BRD) containing proteins involved in fundamental cellular processes, such as meiosis, embryonic development, cell cycle regulation, and transcription, and have elevated activity in human leukemia [132,133,134,135,136,137]. Through their bromodomains, BRD2/RING3 interacts with acetylated histone H4 and BRD4 interacts with acetylated histones H3 and H4, thereby providing a docking station for other proteins to attach to the chromatin [138,139,140,141]. Interaction of BRD2/RING3 has been shown to promote G1/S transition [142]. It has been shown that LANA recruits BRD2/RING3 to chromatin [143,144]. LANA binds to RING3 through the extra-terminal (ET) domain, characteristic of fsh-related proteins, suggesting a highly conserved function in terms of protein-protein interactions [145,146,147]. In *in vitro* assays, it has been shown that BRD2/RING3 mediates phosphorylation of Ser/Thr residues within the *C*-terminal domain of LANA between amino acids 951 and 1107 [143]. RING3 localizes to the euchromatin regions in the interphase nucleus even in the absence of the KSHV viral genome and is released to the cytoplasm during mitosis. In KSHV-infected cells, most of the RING3 proteins co-localize with LANA suggesting that RING3 may contribute to KSHV genome persistence by local euchromatic microenvironment around the viral episomes tethered to the heterochromatic region of the chromosome [144]. The BRD2/RING3 binding domain of LANA was mapped between amino acids 1007 and 1055 [88]. Mutants of LANA capable of supporting replication and dimerization were able to interact with BRD2/RING3, suggesting that RING3 may be important for interaction of the *C*-terminal domain of LANA with heterochromatin [88]. It has also been seen that the DNA binding domain of LANA interacts with the extra-terminal domain of Brd4. Since Brd4 is associated with mitotic chromosomes throughout mitosis and is co-localized with LANA and KSHV episomes on host mitotic chromosomes, Brd4 might also be an interacting protein that mediates LANA tethering onto the mitotic chromosomes [87,88]. A model summarizing the role of cellular proteins in viral genome segregation is presented in Figure 2.

## 6. Role of LANA in KSHV DNA Replication

During latency the KSHV genome exists as a closed circular episome tethered to the host chromosomal DNA and replicates in harmony with the host cellular DNA (once per cell cycle). In general, KSHV-infected PEL cells maintain 50–100 copies of KSHV episome per cell and the copy number remains the same after multiple cell divisions. Viral DNA replication is a fundamental process to maintain a constant number of latent viral genome in the proliferating cells. In the absence of DNA replication, the KSHV genome is rapidly lost from the dividing cells leading to failure in the maintenance of persistent viral infection. LANA, a sequence-specific DNA binding protein, is shown to play an indispensable role in KSHV episomal DNA replication and segregation of the newly constructed genome copies to daughter nuclei. Replication of the KSHV genome is thought to be executed using cellular replication factors and LANA is documented to assist through its interaction with a variety of host cellular proteins.

### 6.1. LANA-TR Mediated DNA Replication of KSHV Episome

The terminal repeats (TRs) in the KSHV genome contain a DNA replication origin *cis*-element called ori-P that consists of two LANA-binding sites (LBS): a higher affinity site/LBS1 and a lower affinity site/LBS2, followed by an adjacent 32-bp GC-rich segment (reviewed in [33]). LANA, being a *trans*-acting protein, binds to the TRs forming a LANA DNA replication element. A 31 bp sequence upstream of LBS1/LBS2 is mapped as a replicator element (RE) and is critical for the initiation of replication. Plasmids containing a single copy of the TR with both LBS1/LBS2 and RE are considered to be replication sufficient in a LANA dependent manner; however, at least two copies of the TR are required for stable episomal persistence. Based on colocalization studies, LANA has been shown to co-localize with the artificial episomes (containing two TR units) along with mitotic chromosomes, thereby providing a model in which LANA docks the KSHV genome to the host chromatin during host cell division. LANA promotes viral DNA replication by directly binding at LANA-binding sites and recruiting the components of cellular pre-replication complexes (pre-RC), which include origin recognition complexes ORC1-6 (ORCs), Cdc6, Cdt1 and mini-chromosomal maintenance proteins (MCMs) to the origin of replication [148,149].

According to the amino acid sequence, LANA is divided into three different protein domains: a proline-rich *N*-terminal domain containing the sequence motif that tethers LANA to host chromosomes, a long glutamic acid-rich internal repeat central domain and a carboxy-terminal domain (amino acids 770–1162 fragment). The association of LANA (LBS1/2 within the TRs) with the pre-RC complex units occurs in a cell cycle dependent manner, facilitated by both the *N*- and *C*-terminal domains of LANA. As only a few viral proteins are expressed during latent infection and these do not posses enzymatic activity required for DNA replication, it is suggested that the LANA-TR complex recruits the cellular DNA replication machinery to replicate viral DNA. LANA’s role in latent DNA replication is regulated through the formation of the LANA-TR complex and further recruitment of the host cell replication machinery in a coordinated manner. Consistent with these studies, mutations within amino acids 4–32 of the *N*-terminal region of LANA and the region that regulates binding of LANA to LANA-binding sites of the TRs, led to a rapid loss of the KSHV genome in human cells, indicating that these regions are critical for DNA replication. Also the *C*-terminal domain of LANA is essential for episomal replication, as it interacts with several chromosome binding and origin recognition complex proteins such as Brd2/RING3/CBP/ORC2 and HBO1 and there is a decrease in the DNA replication efficiency when the expression levels of these proteins are knocked down by siRNAs [150].

**Figure 3:**
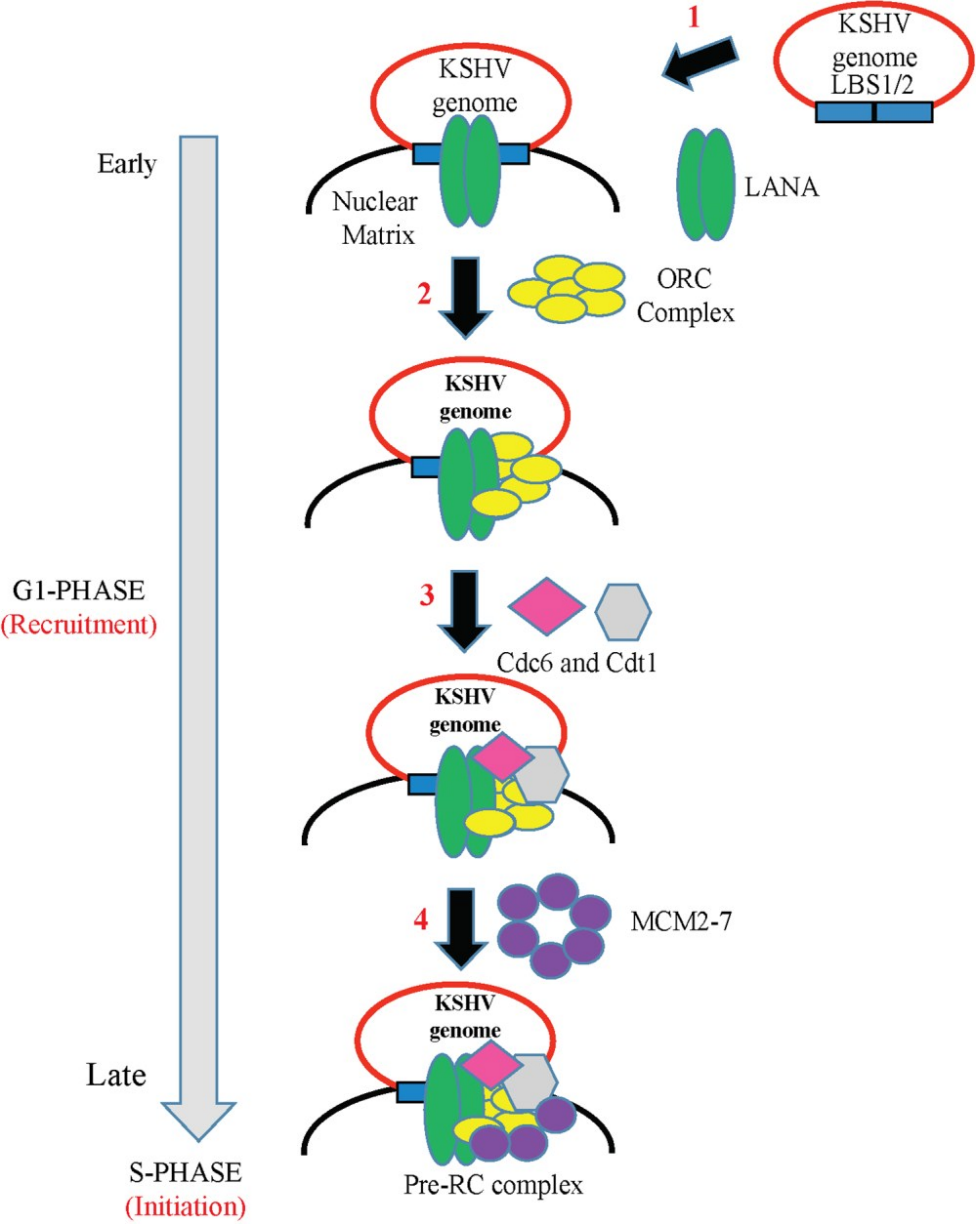
A model of the KSHV latent DNA replication. (**1**) LANA binds to the LANA-binding sites (LBS1 and LBS2) or replication origin of the terminal repeats (TR) region of the KSHV genome and recruits it to the nuclear matrix region; (**2**) LANA then recruits the host cellular machinery factors such as Origin Replication Complexes (ORCs) to the replication origin which is followed by (**3**) sequential loading of Cdc6, Cdt1; and (**4**) heterohexameric complex Mcm2-7 to the origins to form pre-replicative complex (pre-RC) during late G1 phase followed by replication of DNA during early S phase.

The nuclear matrix region has been identified as the replication initiation site for the host cell-cycle dependent viral DNA replication. Studies conducted by Ueda’s group proposed a model for latent DNA replication of KSHV, LANA binds with the nuclear matrix region and the nuclear matrix region serves as the site of the replication factory [33]. According to the proposed model, LANA associates with ori-P through LANA binding sites on TR and recruits it to the nuclear matrix region during the late G1 phase. This LANA-bound ori-P then serves as the launching pad for the recruitment of other cellular proteins, Cdc6, Cdt1 and MCMs (to establish a complete pre-RC complex) to the nuclear matrix region for the initiation of viral DNA replication (Figure 3).

Due to the absence of DNA polymerase/helicase activity by LANA, it is believed that LANA-mediated KSHV DNA replication depends on the enzymes that display these activities. One such enzyme, Topoisomerase II (TopoIIβ), is known to control the topology of DNA and to initiate DNA replication by activating double-stranded breaks on DNA. DNA affinity chromatography and proteomics analysis using KSHV TR DNA and the LANA binding site (as affinity column) recognized topoisomerase IIβ (TopoIIβ) as an important LANA-interacting protein. Our group showed that LANA interacts with TopoIIβ and forms complexes in KSHV-infected cells [151]. These studies confirmed that LANA recruits TopoIIβ to ori-P through its *N*-terminal domain for replication initiation. Further, a selective inhibitor of TopoIIβ, ellipticine, negatively regulated replication of TR confirming its role in DNA replication [151].

A recent report showed that the interaction of LANA with the replication factor C (RFC) complex is critical for KSHV episomal replication and genome persistence [152]. The RFC complex consists of a five-subunit (Rfc1, Rfc2, Rfc3, Rfc4 and Rfc5) protein complex that is needed for DNA replication [153]. The RFC complex is an AAA+ clamp loading ATPase and catalyzes the loading of DNA polymerase clamp loader, PCNA (proliferating cell nuclear antigen) on to the DNA. LANA recruits PCNA and LANA-enhanced PCNA loading is considered to be necessary for viral replication and persistent infection, as LANA mutants lacking RFC interaction negatively regulated LANA-mediated latent DNA replication in infected cells. These findings suggested that PCNA loading is a rate-limiting step in DNA replication and that LANA enhancement of PCNA loading permits efficient virus replication and persistence.

### 6.2. Epigenetic Regulation of LANA-TR Mediated KSHV DNA Replication

It is important to note that epigenetic modifications including DNA methylation, chromatin modifications and nucleosome positioning, also control KSHV DNA replication during latency [43,154]. Chromatin modifications are shown to regulate DNA replication by controlling the recruitment of replication proteins as well as the access of the replication machinery to the KSHV genome. Chromatin structure at the KSHV TR consists of four nucleosomes and two LANA binding sites [86]. The GC rich regions of TR have been shown to have a high tendency to form repressive heterochromatin that alters the binding of the cellular proteins required for pre-RC complex formation [43]. During the G1/S phase of the cell cycle, chromatin structure at the TR is altered and hidden DNA becomes more accessible to replication machinery components. Also, there is significant alteration in histone modification patterns at TR. The TR typically possesses high levels of acetylation at histones H3 and H4, in contrast to the internal region of the genome, which is abundant in activating histone marks (H3K4me3). During G1/S phase, there is a significant reduction in these activating histone marks while histone hyper acetylation remains constant. Levels of H3K4me3 appear to be correlated with the recruitment of MCM3 by the LANA-ORC complex at the latent origin of replication. LANA is also critical to the recruitment of HBO1, a member of the Myst family histone acetyl-transferases that interacts with ORC1 and MCM3 [148].

We recently showed that LANA upregulates the expression of Survivin, an inhibitor of apoptosis to increase the proliferation of KSHV-infected B cells [155]. Our previous studies showed that LANA recruits Aurora kinase B (AK-B), which induces the phosphorylation of Survivin at residue T34 leading to an enhanced activity of p300, which inhibits histone deacetylase 1 (HDAC-1) activity [156]. This in turns leads to an increase in acetylation of histone H3 on the viral genome that further increases the viral copy number in KSHV-infected B cells. The result is a boost of KSHV DNA replication in latently infected B-cells. These studies highlight the innovative role of LANA in the regulation of latent viral replication prior to mitosis.

### 6.3. Non-TR Mediated KSHV DNA Replication

Studies focused on the identification of LANA binding sites on KSHV genome identified TR-associated LANA binding and replication initiation sites. Our continued interrogation of the KSHV genome, led to the discovery of an additional DNA replication origin, called ori-A, using the single molecule analysis of replicated DNA (SMARD) technique [157,158]. In this technique, replicating DNA is labeled with IdU and CldU in order to determine the position, direction and the regions of replication forks on the replicated molecules. Our results indicated that replication of DNA can initiate throughout the KSHV genome. SMARD also showed that the utilization of multiple replication initiation sites occurs across large regions of the genome rather than at a specified sequence. The replication origin of the terminal repeats showed a slight preference for their usage, indicating that LANA dependent origin at the terminal repeats (TR) plays a role in genome duplication. Furthermore, ChIP assays for ORC2 and MCM3, which are part of the pre-replication complex (pre-RC), determined multiple genomic sites with these proteins. These suggested that initiation of replication is likely to be affected by the genomic context rather than by the DNA sequence.

## 7. LANAs Interaction with Various Cellular and Viral Pathways/Promoters

### 7.1. Analysis and Implication of Multiple LANA-Binding Sites within the KSHV Genome

Apart from regulating viral genome copy number and gene expression, LANA is also known to modulate host cell gene expression by interacting with different transcription factors and chromatin regulatory proteins, and through direct binding to the regulatory regions of the cellular genes [88,148,159,160,161,162,163,164,165,166,167,168]. Deep Sequencing and genome-wide analysis has been done to identify the LANA binding sites in KSHV latently infected cells. The results have revealed several LANA binding sites throughout the viral and host-cell genome with a small subset of LANA binding sites near transcription start sites (TSS) and gene promoters [81,169,170] of some cellular genes, suggesting a role for LANA in the regulation of host genes during KSHV infection. Interestingly, LANA binding on host chromatin is seen to be cell type specific and most LANA binding sites are found in endothelial cells [81,169,170].

Two-independent LANA-specific-ChIP-Seq experiments performed using the KSHV-positive BCBL-1 PEL cells have identified a total of 256 LANA binding sites with 17.5% of the peaks (45/256) being situated within ±2 kb of the TSS. In addition, two GC-rich DNA motifs were recognized in these LANA binding sites, with motif 1 being identical to the sequence of KSHV LBS1 and motif 2 displaying less similarity to KSHV LBS1/LBS2. Among the 256 total LANA binding peaks detected, LANA was shown to be associated with the genes within the p53 and tumor necrosis factor (TNF) regulatory networks, both of which help in the survival of latently infected cells. LANA was seen to bind near the TSS [81,170] of numerous genes that interact with p53, suggesting that LANA may be physically and functionally involved with p53 for the co-regulation of a common network of cellular genes important for cell cycle control in response to stress or DNA damage. Based on the comparison of LANA-specific-ChIP-Seq data with IFN-γ–inducible STAT1 DNA binding ChIP-Seq data, Lu *et al.* found that most LANA binding sites either co-localize or lie adjacent to STAT1 binding sites at the promoter regions of STAT1-dependent IFN-γ inducible genes, namely TAP1, PSMB9 and PARL [81]. The most enriched LANA binding sites were seen in the promoter regions of TAP1 and PSMB9, both of which are associated with antigen processing and presentation to the major histocompatibility complex class I (MHC-I) [171]. Indeed, LANA was found to compete with STAT1 for binding to the TAP1/PSMB9 promoter region, thus having the potency to attenuate activation of IFN-γ–inducible genes modulating the host cell antiviral immune response [81].

To better understand the interaction of LANA with cellular chromatin, Don Ganem’s group carried out LANA ChIP-Seq experiments in the KSHV-infected epithelial cell line iSLK-219 (with tight latency and highly inducible lytic reactivation) and KSHV-infected primary lymphatic endothelial cells LEC-219s (phenotypic similarities to KS tumors). These studies suggested that the transcriptionally active promoter sites of host genes targeted by LANA are identical to the LANA binding site 1 (LBS1) motif in KSHV DNA [170]. A total of 267 reproducible LANA-binding sites were observed in iSLK-219 cells and 2481 in LEC-219s. Whereas most of the LANA binding sites in LEC-219 were unique to the LEC-219 cell line, 67% (179 peaks) of the LANA binding sites mapped in iSLK-219 cells were also detected in LEC-219s [170]. While 41.8% of LANA-enriched peaks were located within 1 kb of a TSS, nearly 11.6% of the peaks were also mapped between −1 kb and −10 kb of a TSS (distal promoter). The same study also identified 29 LANA-binding sites in the host genome of BCBL-1 cells, though out of these novel LANA-binding sites only 8 were previously detected and reported in the study by Lu *et al.*, using the same cell line [170]. Additionally, 4 gene promoters (SGMS1, SBF2, IQGAP3 and NIPAL2) were found to bind to LANA in 5 different cell lines studied, namely KSHV-infected endothelial cells (LEC-219), primary blood endothelial cells (BECs), Burkitt’s lymphoma B cells (BJAB), PEL cell line (BCBL-1) and epithelial cells (iSLK-219) [170]. Due to the preferential enrichment of LANA at a TSS, the chromatin that is associated with the proximal promoters occupied by LANA was also examined. ChIP-Seq analysis of histone activating marks (H3K4me3) in iSLK-219 cells displayed the co-localization of LANA and H3K4me3 at proximal promoters, suggesting the possibility of LANA association with active chromatin [170]. Most of the LANA-associated TSS were highly populated with activating H3K4me3 histone marks whereas only a small subset of LANA-enriched regions showed increased levels of the repressive (H3K27me3) histone marks [170]. However, the presence of LANA on the proximal promoter did not impact their transcriptional activity or regulated the deposition of H3K4me3 marks. These observed results were further supported by those reported earlier by Hu *et al.*, where LANA binding to TSS correlated with H3K4me3, but not with the H3K27me3 marks and 86% of all LANA bound promoters were found to be transcriptionally active [169]. Taken together, these results suggested that LANA binding to the host gene promoters in the KSHV-infected cells is not sufficient to modulate the host gene expression, indicating LANA’s indirect role in the regulation of gene expression during infection [169]. The ChIP-Seq results showed that the association of LANA with the host and viral genome was disrupted during late lytic cycle of KSHV infection [170].

LANA Chip-Seq studies performed by Hu *et al.* using BCBL-1 and TIVE-LTC cells (Telomerase-Immortalized human umbilical Vein Endothelial cells) showed that LANA binds to many more promoters in TIVE-LTC cells compared to BCBL-1 (also supported by the study of Mercier *et al*). Additionally, LANA was found to bind to host cellular DNA directly using two distinct motifs [169]. Fewer LANA-binding sites (58 of 2180 LANA binding sites in BCBL-1 cells, and 205 of 2951 LANA-binding sites in TIVE-LTC cells) showed sequence homology to the known LANA binding sites (LBS1/LBS2) [169]. A large number of LANA binding sites instead had a novel motif (TCCAT)_3_ whose affinity for LANA was lower than LBS1, but was comparable to LBS2 [169]. In contrast to the report by Hu *et al*., Mercier *et al.* identified the majority (58.8%) of the LANA ChIP-peaks to have sequences resembling LBS1 [169].

In a recent study by Hu *et al.*, LANA occupancy on the human genome in BCBL-1 and TIVE-LTC cells was studied and 26 genes were found enriched between the two cell types [169]. Three genes, PARL, NIPAL2, IQGAP3 were BCBL-1 specific while four genes, MRPL53, NFYC, CCDC90B and HIST2BE were TIVE-LTC specific. Also WDR74 displayed similar binding profiles in both cell lines [169]. Survivin and Id-1, regulated by LANA, [169,172] contained LANA peaks within the promoters in both cell types. The gene ontology analysis showed that in BCBL-1 cells the putative LANA targets were related to phosphorous metabolic processes, regulation of cellular enzymatic activity and regulation of cellular response to stress, whereas in TIVE-LTC cells the LANA targets detected are involved in regulation of macromolecular metabolic process, nutrient levels, and angiogenesis which is a hallmark of KS [169]. However, it was observed that 14% of the cellular promoters that are bound by LANA were not expressed in BCBL-1 cells during latency [169]. In KSHV-infected LEC cells, only 3.7% of the host gene promoters bound by LANA were differentially regulated [170]. This suggested that LANA binding to a host gene promoter alone has no direct impact on gene expression. However, certain genes like IQGAP3 gene can be directly regulated by LANA [81,170]. Thus, it appears that although LANA binding to the host gene promoter is not responsible for the host gene expression, it still remains an important permissive factor that allows co-ordination with other factors to regulate host gene expression.

### 7.2. LANA Associates with Several Viral Promoters and Causes Epigenetic Modifications

Upon *de novo* KSHV infection, there is a constant expression of the key latent genes along with a transient yet robust expression of a handful of lytic genes. A series of studies have shown that the immediate-early lytic protein, Replication and Transcription Activator (RTA), encoded by KSHV ORF50 (691 amino acid residues/120 kDa) is the only viral lytic protein that is necessary and sufficient to disrupt latency and promote the complete lytic cascade. RTA protein acts as the master latent-to-lytic switch that triggers KSHV to enter into the productive transcriptional program required for viral spread and KS pathogenesis (reviewed in [173]). RTA auto-activates its own promoter and trans-activates the expression of multiple downstream lytic genes including ORF K8, K5, K2, K12, ORF 6, ORF 57, ORF 74, K9, ORF 59, K3, ORF 37, K1, K8.1A, ORF 21, vIL-6, PAN RNA, vIRF1, ORF-K1 and small viral capsid proteins ORF 65, either alone through an RTA-responsive element or in combination with other viral regulatory genes [69,174,175,176]. KSHV RTA was also shown to interact with host cellular proteins and modulate cellular as well as viral gene expression. However, establishment of latency disrupts a full-blown expression of RTA and other lytic genes, though the molecular events behind this rapid inhibition of the lytic promoters are still poorly understood.

Earlier study showed a significant increase in the immediate-early gene expression following transfection of a LANA-deletion mutant of KSHV, suggesting that LANA plays a critical role in transcription repression of the lytic genes [177]. LANA, the major latent protein, is involved in the repression of the basal level of RTA promoter activity and other RTA-responsive lytic promoters as: (1) LANA interacts with several transcription repressors and histone-modifying enzymes associated with lytic genes silencing (reviewed in [173]); (2) LANA promotes establishment of latent histone modification patterns on the RTA promoter and represses the RTA-mediated gene activation (reviewed in [69]); and (3) The mutual LANA and RTA feedback regulatory mechanism promotes the establishment of KSHV latency [162].

The transcription repression activity of LANA occurs through its interaction with transcriptional repressors (heterochromatin protein HP1α, methyl-CpG-binding protein MeCP2, histone deacetylase co-repressor mSin3 complex and DNA methyltransferases, DNMTs) and chromatin-remodeling proteins (H3K9me3 histone methyltransferase SUV39H1, H3K9 demethylase KDM3A, histone acetyltransferase CBP and chromatin transcription complex FACT) [86,161,178,179,180,181,182]. Previous reports have indicated that interaction between LANA and the recombination signal sequence binding protein Jκ (RBP-Jκ), a major transcriptional repressor of the Notch signaling pathway, is essential to suppress the transcription of RTA [183,184]. By directly interacting with RBP-Jκ protein, LANA not only recruits co-repressors to down-regulate the expression of RTA gene but also represses RTA auto-activation activity. The fact that both positive and negative regulators of RTA gene expression use the same RBP-Jκ dependent mechanism, suggests that the switch between latency and lytic reactivation is fine-tuned by the levels of LANA and RTA proteins in KSHV-infected cells. Furthermore, RTA also induces LANA expression providing a negative feedback in keeping viral lytic reactivation under check.

DNA methylation and post-translational histone modifications play a central role in the regulation of gene expression [185]. DNA methylation of functionally conserved immediate early gene of herpesvirus genome by *de novo* methyltransferases, DNMT3a/DNMT3b results in establishment of methylation marks on the immediate early gene promoter (but not at the latency locus) [186], followed by gene silencing (reviewed in [34]). Treatment of BCBL-1 cells with histone deacetylase inhibitors, including sodium butyrate (NaB) and Trichostatin A, causes a rapid dissociation of LANA from the RTA promoter, initiating transcription activation of the RTA gene [187]. Interestingly, expression of LANA is also regulated by post-translational modifications such as arginine methylation, phosphorylation and SUMOylation [178]. Furthermore, phosphorylation of LANA by several kinases including glycogen synthase kinase (GSK-3), DNA-PK/Ku and Pim family kinase members, Pim-1 and Pim-3, has been reported to promote viral reactivation by negative modulation of LANA function [188,189,190]. LANA is also identified as a substrate for protein arginine methyltransferase 1 (PRMT1) and methylation at the R20 site is found to influence strong binding of LANA to the KSHV genome and repression of lytic genes [191]. Hence, these studies clearly show that LANA plays a role in lytic gene silencing during the establishment of latency.

Several independent groups have characterized the epigenetic modification of KSHV episomes during latency using genome-wide ChIP-seq assays, which revealed that latency-associated genes such as LANA are distinctively associated with activating histone marks (acetylated H3 and H3K4me3) while most lytic genes are enriched with either bivalent chromatin (acH3, H3K4me3 and H3K27me3) or active chromatin (acH3/H3K4me3-rich) [192,193,194,195]. In addition, two histone-modifying enzymes, EZH2, a H3K27me3 histone methyltransferase of the Polycomb Repressive Complex 2 (PRC2) and JMJD2A, a H3K9me3 histone demethylase, are associated with the latent KSHV genome [196,197]. The regulated removal of the gene-silencing epigenetic mark, trimethylation of lysine 27 of histone H3 (H3K27me3), by either transient expression of UTX or JMJD3 (the H3K27 demethylase) or by blocking with EZH2 (H3K7 methyltransferase) of PRC2 complex, disrupts latency and induces lytic reactivation [194]. Intriguingly, LANA can associate with JMJD3 and EZH2, and this interaction maintains H3K27me3-enriched heterochromatin on lytic genes to repress their expression during latency [194].

### 7.3. LANA Associates with Host Proteins and Signaling Pathways

LANA is shown to interact with various cellular proteins, indicating that LANA may target these proteins to promote KSHV-mediated tumorigenesis. Although KSHV is equipped to manipulate and deregulate several cellular proteins and associated signaling pathways (reviewed in [38,198]), it is not yet understood how this interaction inhibits apoptosis and leads to cell proliferation and cell transformation. Deeper understanding of the interplay of LANA and cellular factors in KSHV-infected cells may provide valuable information on the precise mechanisms of KS infection that could enable the development of drug therapies for KSHV-induced oncogenesis.

At the gene transcriptional level, LANA has been shown to inhibit KSHV lytic reactivation as it blocks the expression of KSHV RTA, which is critical for the KSHV latency to lytic switch [176]. LANA has been reported to display both transcription repression and activation activities by interacting with multiple cellular transcriptional factors, including the components of mSin3 complex, CBP, RING3, GSK-3β, p53 and pRb (for gene silencing) and E2F, Sp1, RBP-Jκ, ATF4/CREB2, CBP, Id-1 and Ets-1 (for gene activation) [160,161,162,172,199,200,201,202,203,204,205]. Here we discuss some of these key cellular proteins along with newly identified LANA-cellular protein interactions.

#### 7.3.1. LANA Interaction with p53 and pRb

LANA has long been known to bind to and block p53-transcriptional activity and inhibit p53-induced cell death [156,166]. In human cells, constitutive expression of LANA was shown to induce chromosomal instability by suppressing the transcription of p53 from its endogenous promoter. Our previous studies have indicated that in KSHV-infected cells, p53 can be degraded by recruitment of the cellular EC_5_S ubiquitin complex, targeted by the SOCS-box (suppressor of cytokine signaling) motif of LANA [206]. Recently, our group reported that LANA can also upregulate the levels of Aurora A, a centrosome-associated Ser/Thr oncogenic kinase, which promotes phosphorylation of p53 and LANA-mediated p53 ubiquitylation and degradation [207]. LANA was also shown to interact with the retinoblastoma (Rb) protein and enhance the transcriptional activation of the E2F-responsive gene/cyclic E promoter [208]. Co-expression of LANA and H-Ras has been shown to transform primary rat embryonic fibroblast (REF) cells *in vitro* [166]. LANA expression in lymphoid cells is found to overcome the cyclin-dependent kinase inhibitor, p16INK4a, and BRD4- and BRD2/RING3-induced G1 cell cycle arrest and stimulate E2F-mediated S-phase entry [62,88].

#### 7.3.2. LANA Interaction with GSK-3β (Wnt Signaling Pathway)

LANA, also interacts with glycogen synthase kinase (GSK-3β), a kinase involved in phosphorylation and subsequent degradation of many proteins involved in cell cycle regulation, such as β-catenin, proto-oncoprotein c-Myc, c-Jun and cyclin D genes [209]. Interaction of LANA with GSK-3β, an important modulator of the Wnt signaling pathway, leads to an overall inactivation and nuclear sequestering of GSK-3β due to extracellular signal-regulated kinase 1/2 (ERK1/2)-mediated phosphorylation of GSK-3 at Ser9, which stabilizes cytosolic β-catenin and makes it available for transcriptional activation of target genes [210]. LANA also interacts and stabilizes c-Myc, another known GSK-3β substrate whose activity is regulated by ERK1/2 [211]. LANA decreases c-Myc phosphorylation on Thr58, an event that promotes Myc-induced apoptosis; and increases phosphorylation of c-Myc at Ser62, an event that transcriptionally activates c-Myc [211].

#### 7.3.3. LANA Interacts with ANG and Promotes Angiogenesis

LANA has been reported to interact with the 14-kDa multifunctional protein angiogenin (ANG), which is considered to play a critical role in establishment of KSHV latency, anti-apoptosis and tumor angiogenesis [212]. Also, in KSHV-latently infected telomerase-immortalized microvascular endothelial (TIME) and BCBL-1 PEL cell lines, LANA and ANG were found to co-interact with Annexin A2, a protein that plays significant roles in cell proliferation, apoptosis and exo-/endocytosis [213]. Suppression of Annexin A2 or ANG expression in PEL cells was found to increase cell death, whereas depletion of Annexin A2 led to a concomitant decrease in both ANG and LANA protein expression, indicating that these three proteins are integrated and functionally important to promote viability of latently infected cells.

#### 7.3.4. LANA Interacts with BMP-Activated Smad1

Recently, in the KSHV-transformed mesenchymal precursor (KMM) cells, KSHV LANA was shown to affect the bone morphogenetic protein (BMP) signaling pathway and convert it to an oncogenic BMP-Smad1-Id pathway, which might contribute to the pathogenesis of KSHV-induced malignancies [200]. BMP signaling pathways are involved in both cell promotion and inhibition of tumor progression. Also, inhibitor of DNA (Id) proteins are major downstream targets of BMP signaling and negative-regulators of basic helix-loop-helix (HLH) proteins [214]. In these studies, the *N*-terminus of LANA (1–432 amino acid residues) is found to interact with MH2-M, the LANA-binding domain of BMP-activated pSmad1 [199]. LANA-Smad1 interaction led to an increase in the Smad1 loading on the Id promoter which then upregulates the Id protein expression in the KS lesions. Chemical inhibition of the BMP-Smad1-Id pathway by Dorsomorphin and WSS25 inhibits the growth of the KSHV-induced tumors, indicating that small inhibitors targeting this pathway can serve as potential therapeutic agents for the treatment of KS.

#### 7.3.5. LANA Interacts with Host KAP1 and Facilitates the Establishment of KSHV Latency

Two studies have recently reported that LANA interacts with a new host nuclear protein, called KAP1 (KRAB-associated protein 1) both *in vivo* and *in vitro* and down-regulates the lytic gene expression to facilitate KSHV latency [215,216,217]. KAP1 is a cellular transcriptional repressor that controls chromatin remodeling and KSHV biphasic life cycles. LANA is reported to bind to both the *N*- and *C*-terminal domains of KAP1, which interacts with SETDB1, H3K9me3 methyltransferase and recruits them to the lytic promoter region of the KSHV genome [218]. In cells harboring latent KSHV, shRNA knockdown of KAP-1 resulted in the induction of lytic genes and 5-fold increase of RTA-mediated lytic reactivation. Thus, KAP1 is believed to play an important role in the complete shutdown of the transient lytic gene expression during the early stages of KS infection.

#### 7.3.6. LANA Interacts with Daxx and Contributes to VEGFR–Mediated Angiogenesis

The death-associated protein Daxx is a multifunctional protein that binds to several cellular host proteins and regulates a variety of cellular processes, including transcriptional repression and apoptosis [219]. Co-immunoprecipitation analysis of human HeLa cells harboring KSHV LANA identified Daxx as a potential LANA-binding protein [202]. These results indicate that LANA associates with Daxx and colocalizes in the nucleus of KSHV-infected BCBL-1 cells. The Glu/Asp-rich domain within LANA (321–344 amino acid region of LANA) was shown to bind to the central 63–440 amino acid region within Daxx, containing two paired amphipathic helices and its following 20 amino acids. Also, Daxx was found to repress Ets-1 dependent VEGF receptor-1 gene expression. However, there is an indirect regulation of the Ets-1 responsive promoters through a LANA-Daxx interaction [202]. Co-IP and immunoblotting experiments indicate that LANA-binding to Daxx disrupts the association between Daxx and Ets-1, which in turn, contributes to an increased level of VEGR receptor in KS tissues [202]. Vascular endothelial growth factor (VEGF) and its receptors are highly expressed in KS lesions and are known to play a key role in angiogenesis.

## 8. Concluding Remarks

The viral protein LANA encoded by KSHV during latent infection is critical for the maintenance of latency, episome replication, segregation, and also for regulating certain viral and cellular genes. LANA has been reported to interact with many host cellular proteins important for DNA replication, transcriptional regulation and cell cycle control. The interaction between LANA and host proteins leads to maintenance of the KSHV episome and transformation of the host cell. Strategies that can interrupt the interaction between LANA and host cell proteins may provide effective treatment and prevention of KSHV-associated diseases. LANA is also known to regulate several host cellular pathways. ChIP-Seq data revealed that LANA binds directly to the transcription start site of many host cellular genes. Interestingly LANA was found to bind to the genes within the p53 and TNF networks, both of which promote survival of latently infected cells. However, only a small percentage of LANA-bound cellular promoters showed direct LANA-driven gene regulation. Therefore, LANA binding to the promoter region of host genes may facilitate participation of other factors to regulate the LANA-driven host gene expression. Further investigations of LANA’s role in KSHV latent DNA replication, manipulation of host and viral gene expression, and association with several cellular and viral proteins are required to facilitate our understanding of LANA-mediated KSHV latency and related oncogenesis.
